# Hypertensive Diabetic Patients: Casual Pulse Pressure and Ambulatory Blood Pressure Monitoring (ABPM) as Superior Predictors of Future Cardiovascular Events

**DOI:** 10.7759/cureus.77974

**Published:** 2025-01-25

**Authors:** Inês S Pinheiro, Adriana Pacheco, Simão Carvalho, Tiago Aguiar, José Mesquita Bastos

**Affiliations:** 1 Internal Medicine, Unidade Local de Saúde da Região de Aveiro, Aveiro, PRT; 2 Cardiology, Unidade Local de Saúde da Região de Aveiro, Aveiro, PRT

**Keywords:** ambulatory blood pressure monitoring, cardiovascular events, diabetes, hypertension, pulse pressure

## Abstract

Hypertensive (HTN) patients with type 2 diabetes mellitus (T2DM) are at an increased risk of adverse survival outcomes compared to non-diabetic individuals. This study aimed to retrospectively evaluate the prognostic significance of ambulatory blood pressure monitoring (ABPM) in a subgroup of diabetic patients. A total of 823 HTN patients, followed since 1994 at a hospital (follow-up: 11.8 ± 5.6 years), were included in the study. These patients underwent ABPM using a SpaceLabs 90207 device (SpaceLabs Healthcare, Snoqualmie, WA, USA) during a normal working day. Data from both the ABPM and office blood pressure (BP) values, as well as cardiovascular risk factors, were analysed. The patients were divided into two groups: diabetic (n = 240) and non-diabetic (n = 583). Data were analysed using IBM SPSS Statistics for Windows, Version 25 (Released 2017; IBM Corp., Armonk, NY, USA). Our study showed that HTN patients with T2DM experienced more severe cardiovascular events (χ² = 25.34, p < 0.001), heart failure (χ² = 27.7, p < 0.001), and mortality (χ² = 11.8, p < 0.01). Also, they had elevated pulse pressure (PP) values (analysed either as a continuous variable or using a 60-mmHg cut-off within ABPM values), which were associated with worse survival outcomes. In the analysis of HTN phenotypes, the presence of resistant hypertension (RH) was significantly higher in the diabetic group (χ² = 8.14, p < 0.05), which is associated with poorer survival. Despite the growing body of research, there are currently no studies in the literature using ABPM data specifically in diabetic patients. These data could offer valuable insights into the BP patterns of these patients, helping to define the most effective therapeutic strategies.

## Introduction

Type 2 diabetes mellitus (T2DM) is a well-established condition associated with an increased risk of developing hypertension (HTN), while HTN is also recognized as a significant risk factor for the development of diabetes [1]. The Framingham Heart Study demonstrated that T2DM is linked to a two- to four-fold increased risk of HTN, peripheral arterial disease, and myocardial infarction. Similarly, the CARDIA (Coronary Artery Risk Development in Young Adults) Study identified HTN as a precursor to diabetes development, emphasizing the bidirectional relationship between these conditions [2,3].

The pathophysiological mechanisms linking HTN and diabetes are predominantly driven by insulin resistance. This process contributes to arterial stiffening, which is associated with elevated systolic blood pressure (SBP) and pulse pressure (PP). Additionally, the maintenance of HTN contributes to vascular remodelling and reduced arterial compliance, which further exacerbates arterial stiffness [4,5].

For instance, a study analysing arterial stiffness indices and glucose metabolism in a biracial cohort of 4,701 individuals aged 45 to 64 revealed that patients with borderline abnormal glucose tolerance or T2DM exhibited greater arterial stiffness compared to those with normal glucose tolerance [6]. These findings propose that elevated glucose and insulin levels may synergistically intensify arterial stiffness, playing a critical role in the early pathophysiology of HTN and cardiovascular disease in T2DM [4,6-9].

Ambulatory blood pressure monitoring (ABPM) is well-established as a superior method for assessing BP variability and provides a more accurate correlation with target organ damage in HTN patients [10]. In diabetic populations, however, significant gaps remain in understanding how ABPM findings can guide clinical interventions and predict outcomes.

This study aimed to longitudinally and retrospectively evaluate the prognostic significance of ABPM in a subgroup of diabetic patients, addressing the need for more targeted research in this area. By investigating the role of ABPM in this context, the study seeks to provide a deeper understanding of BP dynamics and their implications for managing HTN and reducing cardiovascular risk in individuals with T2DM.

## Materials and methods

Study population

This study included a cohort of 823 patients with HTN who had been followed since 1994 at a hospital. The time of follow-up was 11.8 ± 5.6 years. The inclusion criteria encompassed patients with confirmed HTN who had undergone ABPM. Those who had not undergone ABPM or whose HTN diagnosis was not confirmed during the consultation were excluded.

ABPM

ABPM was conducted using the Spacelabs 90207 (SpaceLabs Healthcare, Snoqualmie, WA, USA) device, and its accuracy was validated by previous studies [11,12]. BP measurements were recorded over a 24-hour period on a typical working day. Patients were instructed to maintain their regular daily activities during the monitoring period. The nighttime period was self-reported by patients in a sleep diary and subsequently validated during the interpretation of the results.

Office BP measurements

In addition to ABPM, office BP values were recorded during routine clinical visits. Both systolic and diastolic BP readings were taken according to established guidelines [13], ensuring proper cuff size, patient positioning, and resting time prior to measurement.

Additional clinical data collected

Other clinical characteristics were also collected, such as age, weight, body mass index (BMI), and comorbidities, including dyslipidemia, previous cardiovascular events, heart failure, and mortality.

Group classification

The patients were stratified into two primary groups based on comorbidity status: diabetic and non-diabetic. Further subgroup phenotypes were defined based on BP control and treatment response as follows: (i) resistant hypertension (RH) (n = 103), defined as BP ≥130/80 mmHg over 24 hours on ABPM, despite treatment with three or more anti-HTN medications at maximum tolerated doses, including a diuretic; (ii) non-resistant hypertension (NRH) (n = 375), patients on three or more anti-HTN medications at maximum tolerated doses, including a diuretic, who met RH criteria in office BP but not on ABPM; (iii) white coat resistant hypertension (WCRH) (n = 284), patients with office BP readings meeting HTN criteria but normal values on ABPM; (iv) controlled hypertension (CH) (n = 106), patients whose BP was controlled with two anti-HTN medications in both office and ABPM measurements.

Statistical analysis

Data were analysed using IBM SPSS Statistics for Windows, Version 25 (Released 2017; IBM Corp., Armonk, NY, USA). For continuous variables with normal distribution, parametric tests, including the Independent Samples t-test, were applied. Non-parametric variables were analysed using the Chi-square test. Survival analysis was performed using Kaplan-Meier event-free survival curves to evaluate outcomes over time. Additionally, univariate and multivariate Cox regression analyses were conducted to identify predictors of adverse events and to evaluate the impact of clinical and demographic variables on outcomes.

## Results

The subgroup of diabetic HTN patients consisted of 240 patients, with an average age of 61.2 ± 10.3 years, 45.4% of whom were female. In the non-diabetic group (n = 583), the average age was 56.6 ± 12.2 years, with 49.3% being female.

**Figure 1:**
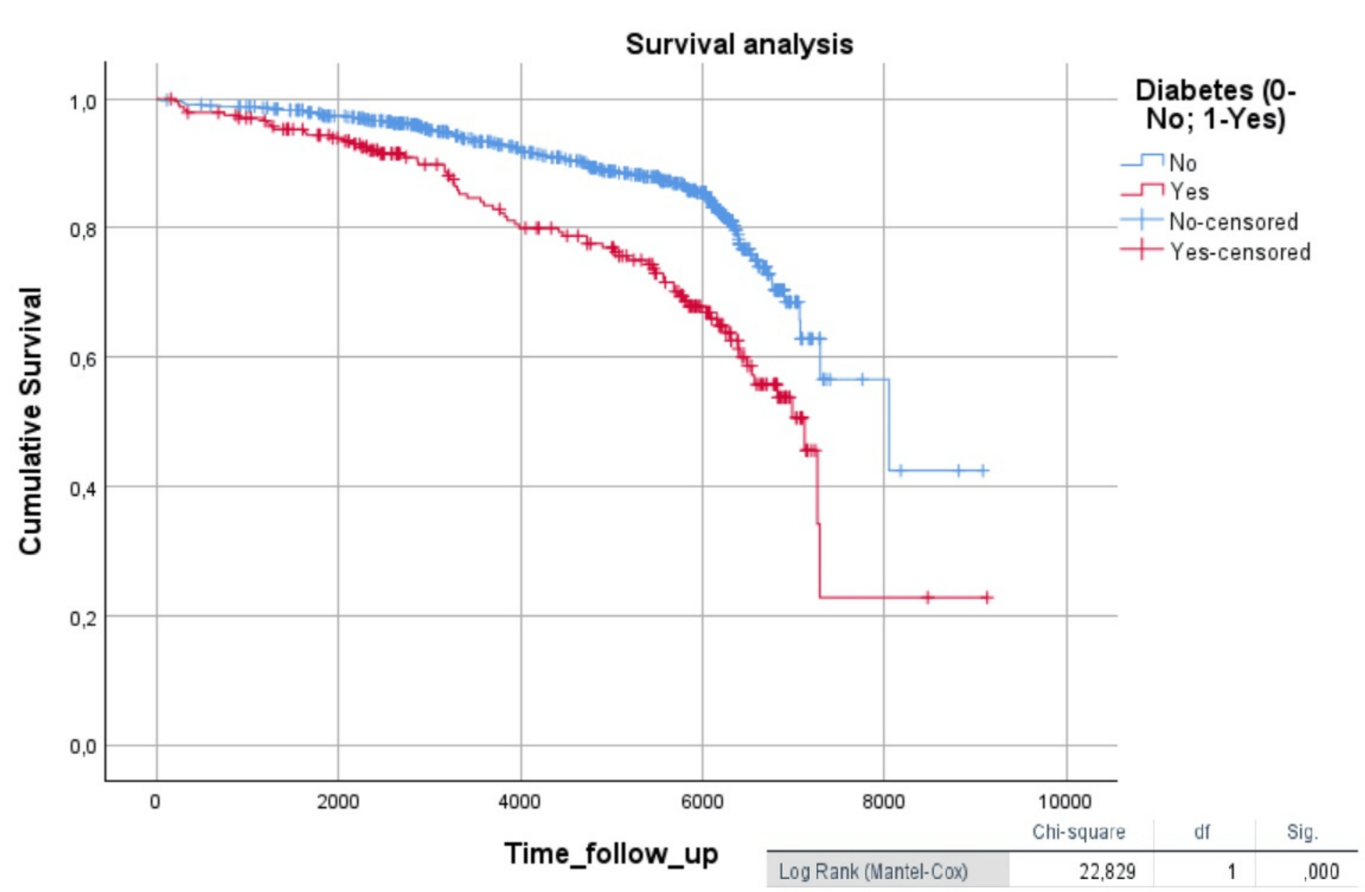
Kaplan-Meier survival curve comparing diabetic patients to non-diabetic patients The Kaplan-Meier curve graphically represents the survival function (time until death or the occurrence of a cardiovascular event). Time is plotted on the x-axis, and the survival rate is plotted on the y-axis.

Diabetic patients had a worse Kaplan-Meier hypertensive event-free survival curve compared to non-diabetic patients (log-rank = 22.9, p < 0.001) (Figure 1).

In the exploratory analysis of the two groups (diabetic vs. non-diabetic), using a Student’s t-test, statistically significant differences were found in age, weight, BMI, casual diastolic blood pressure (DBP), 24-hour SBP, and 24-hour DBP. The PP for both casual measurements and ABPM during the daytime and nighttime also showed statistical significance between the two groups, with diabetic patients having higher values (p < 0.05) (Table 1).

**Table 1:** Exploratory analysis of the two groups (Student’s t-test) BMI: Body Mass Index; DBP: Diastolic Blood Pressure; SBP: Systolic Blood Pressure; PP: Pulse Pressure

	Diabetic patients	Non-diabetic patients	Student’s t-test, p-value (<0.05)
Age (years)	61.92 ± 10.37	56.64 ± 12.22	0.001
Weight (kg)	80.42 ± 13.798	73.73 ± 13.33	0.000
BMI (kg/m^2^)	29.98 ± 5.269	27.33 ± 4.37	0.000
DBP casual (mmHg)	93.98 ± 15.01	96.63 ± 14.45	0.019
SBP 24h (mmHg)	134.14 ± 15.70	131.65 ± 15.87	0.04
DBP 24h (mmHg)	77.80 ± 10.13	80.83 ± 11.09	0.000
PP casual (mmHg)	61.58 ± 19.07	57.49 ± 17.61	0.003
PP 24h (mmHg)	56.37 ± 12.21	51.02 ± 10.23	0.000
Daytime PP (mmHg)	56.90 ± 12.30	51.45 ± 10.52	0.000
Nighttime PP (mmHg)	55.70 ± 13.07	50.23 ± 10.50	0.000

Diabetic patients also had a higher prevalence of previous cardiovascular events (χ² = 25.34, p < 0.001), heart failure (χ² = 27.7, p < 0.001), and mortality (χ² = 11.8, p < 0.01) (Table 2).

**Table 2:** Exploratory analysis of the two groups (Chi-square test) CV: Cardiovascular

	Diabetic patients	Non-diabetic patients	χ2	p-value
CV events	59.30%	40.70%	25.34	<0.001
Heart failure	67.60%	32.40%	27.67	<0.001
Death	70.00%	30.00%	11.81	0.001

**Figure 2:**
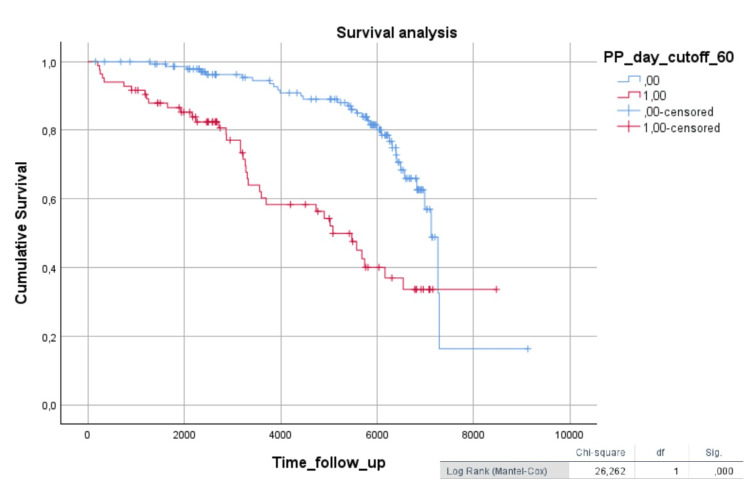
Kaplan-Meier survival analysis in diabetic patients, comparing 24-hour PP (cutoff 60 mmHg) The Kaplan-Meier curve graphically represents the survival function (time until death or the time of a cardiovascular event). Time is plotted on the x-axis, and the survival rate is plotted on the y-axis. PP: Pulse Pressure

**Figure 3:**
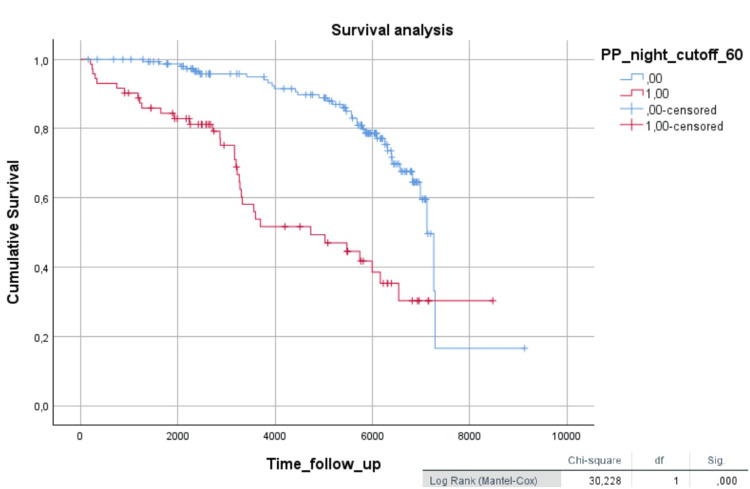
Kaplan-Meier survival analysis in diabetic patients, comparing nighttime PP (cutoff 60 mmHg) The Kaplan-Meier curve graphically represents the survival function (time until death or the time of a cardiovascular event). Time is plotted on the x-axis, and the survival rate is plotted on the y-axis. PP: Pulse Pressure

In the Kaplan-Meier survival analysis, diabetic patients with 24-hour PP values above 60 mmHg (Figure 2) and nighttime PP values above 60 mmHg (Figure 3) showed worse survival (log-rank = 26.2, p < 0.001; log-rank = 30.2, p < 0.001, respectively).

In the multivariate Cox analysis, adjusted for confounding variables (age, weight, sex, casual SBP, casual PP, dyslipidemia, and previous cardiovascular events), the 24-hour SBP, nighttime SBP, nighttime PP, 24-hour PP with a cut-off >60 mmHg, and nighttime PP with a cut-off >60 mmHg were significant negative predictive independent factors for the occurrence of an event (Table 3).

**Table 3:** Multivariate analysis - Cox regression SBP: Systolic Blood Pressure; PP: Pulse pressure

	HR (95% CI)	Cox regression, p-value (<0.05)
SBP 24h	1.04 (1.00-1.10)	<0.05
Night SBP	1.03 (1.00-1.06)	<0.05
Night PP	1.05 (1.05-1.09)	<0.05
PP 24h >60mmHg	4.65 (1.26-17.1)	<0.05
Night PP >60mmHg	6.70 (1.68-26.7)	<0.01

**Figure 4:**
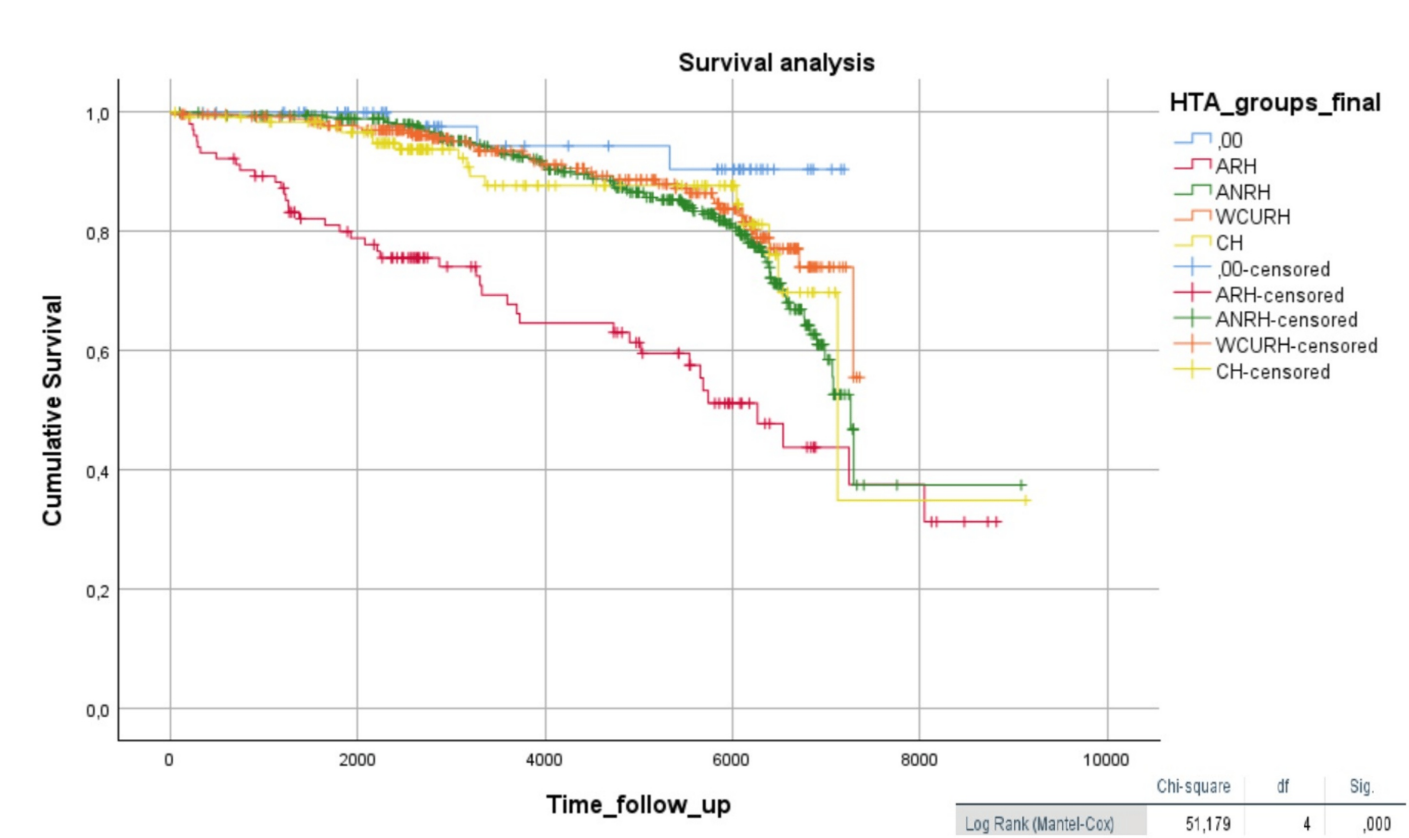
Kaplan-Meier analysis of hypertension phenotypes The Kaplan-Meier curve graphically represents the survival function (time until death or the time of a cardiovascular event). Time is plotted on the x-axis, and the survival rate is plotted on the y-axis. RH: Resistant Hypertension; NRH: Non-resistant Hypertension; WCRH: White Coat Resistant Hypertension; CH: Controlled Hypertension

In the analysis of HTN phenotypes (RH, NRH, WCRH, and CH), the presence of RH was significantly higher in the diabetic group (χ² = 8.14, p < 0.05). It is worth noting that, in the analysis of the total sample (n = 823), patients with RH exhibited worse survival compared to the other groups (log-rank = 51.2, p < 0.001) (Figure 4).

## Discussion

Our results showed that HTN patients with T2DM experience worse survival outcomes compared to non-diabetic patients. Furthermore, patients with T2DM more commonly present with RH, which is associated with poorer survival [14].

Also, the findings of this study reinforce the prognostic value of PP, particularly as measured through ABPM, in patients with HTN and T2DM. Elevated PP values (analysed either as a continuous variable or using a 60-mmHg cut-off within ABPM values), especially during the nighttime period, were associated with worse cardiovascular outcomes. In the literature, high PP is associated with an increased risk of death, cardiovascular events, stroke, chronic kidney disease, and retinopathy [15,16]. The reason why nighttime PP is an independent predictor of total mortality remains unknown, but there are other studies that have had similar results [17].

The literature further mentions that, in diabetic patients, PP has a predictive value for the occurrence of cardiovascular events, and PP >60 mmHg in elderly diabetic patients may increase the risk of cardiovascular events [9,18,19].

These results underscore the importance of incorporating PP into cardiovascular risk assessment for these patients, particularly in high-risk subgroups, such as those with RH.

Moreover, the results highlight that ABPM not only improves BP evaluation in diabetic patients but also provides more accurate insights into circadian PP patterns, which may assist in tailoring therapeutic strategies. Recent studies suggest that specific interventions, such as the use of SGLT2 inhibitors, can significantly reduce BP, offering additional cardiovascular benefits [20]. For instance, empagliflozin has demonstrated the ability to lower 24-hour PP, comparable to conventional anti-HTN therapies, while also reducing the risk of heart failure and cardiovascular mortality [21,22]. In the future, studies may be conducted to evaluate the impact of these drugs on ABPM.

It is noteworthy, however, that gaps remain in the literature regarding the impact of various antidiabetic regimens on BP and PP. Future studies should explore the interplay between glucose levels, insulin resistance, and arterial stiffness to identify more effective therapeutic strategies for preventing cardiovascular events in T2DM patients.

Finally, this study emphasizes the necessity of a multidisciplinary approach to managing these patients, combining pharmacological and non-pharmacological interventions to optimize cardiovascular outcomes.

It is important to take into consideration the limitations of this study: it is a retrospective study with a sample from a single hospital, and other variables could have been assessed, such as other comorbidities.

## Conclusions

The findings highlight the increased risk of poorer survival outcomes in HTN patients with T2DM, particularly those with RH. These results emphasize the need for targeted interventions to improve patient prognosis.

Furthermore, to our knowledge, there are currently no studies in the literature using ABPM data specifically in diabetic patients, which could provide valuable insights into the BP patterns of these patients and help define the best therapeutic strategies.
